# Theoretical and retrograde calculation of blood alcohol: wide estimates using the Academy Standards Board for calculations over an extended period of time

**DOI:** 10.1093/labmed/lmaf055

**Published:** 2025-09-18

**Authors:** Alan H B Wu, Neal Benowitz

**Affiliations:** Department of Laboratory Medicine, University of California San Francisco, San Francisco, CA, United States; Department of Medicine, University of California San Francisco, San Francisco, CA, United States

**Keywords:** blood alcohol concentrations, Widmark equation, volume of distribution

## Abstract

**Introduction:**

Toxicologists defend alcohol testing from their laboratories for medicolegal cases.

**Methods:**

A 22-year-old woman drank 144.1 g of alcohol over 8.25 hours at work. Precise details of her drinking (time, volume, and alcohol content) were provided. At her shift’s end, her supervisor allowed her to drive home, during which time she caused the death of another driver. Her blood alcohol concentration (BAC) was 2.20 g/L, tested a few hours later. She did not drink alcohol after her work discharge. The decedent’s family sued her employer for failure to recognize intoxication. An expert was engaged to perform both theoretical and retrograde BAC estimates based on consumption and the reported BAC, respectively.

**Results:**

Blood alcohol concentration calculations require use of the volume of distribution and the alcohol metabolism rate. The Academy Standards Board recommends reporting a range of BAC calculations. The theoretical and retrograde calculations in this case largely matched each other, suggesting that the driver did not drink after work. Due to the long interval between alcohol intake and her work dismissal, the theoretical calculation produced a wide BAC range (1.4-4.6 g/L). The retrograde calculation range was tighter (2.6-3.1 g/L).

**Discussion:**

A prolonged duration between drinking and testing produces a range of BAC results that can cause ambiguities in legal proceedings.

## Introduction

Blood alcohol concentration (BAC) is an essential part of many criminal cases and civil lawsuits, especially relating to assaults or motor vehicle accidents. When BAC testing data are available, forensic experts are often engaged to “back”-estimate the BAC (retrograde) to some earlier moment in time where the signature event took place. During the postabsorptive phase (ie, after all consumed alcohol has been absorbed from the stomach), ethanol exhibits zero-order metabolism.^1^ Therefore, a constant rate of metabolism, termed “β,” is expressed in units of percentage alcohol declined per hour.

Forensic experts may also be engaged to “forward”-estimate the BAC (theoretical) after drinking to some future moment in time. Calculations are made given assumptions as to the amount of alcohol consumed, the ethanol percentage from those drinks, and the time of consumption by the individual in question. Estimates of postabsorptive blood alcohol levels are computed using the Widmark formula,^2^ where


$$\begin{array}{l} BAC\;=\;alcohol\;dose\;(mg)/VD\;(L), \end{array}$$

where *Vd* is the volume of distribution for ethanol expressed in liters per kilogram of body weight.

The rate of ethanol metabolism is also used if time has lapsed between the end of drinking and the sentinel event. In both situations, an estimated BAC is used by counsel and jurists when drawing conclusions about the likelihood of impairment.

The American Academy of Forensic Sciences Academy Standards Board (ASB) has published recommendations for performing alcohol calculations in forensic cases.^3^ For theoretical calculations (predicting a BAC from drinking history), the ASB recommends using a range of Vd from 0.45 to 0.81 L/kg if the sex is unspecified, 0.58 to 0.83 L/kg for male individuals, and 0.43 to 0.73 L/kg for female individuals. If the person in question’s height and weight are known, the total body water (TBW) can be calculated, thereby narrowing the range of Vd. The ASB recommends the following equation for male and female individual:


$$\begin{array}{lll} TBW\;\left(females\right)\;= & \;2.097+\left(0.1069\;\times \;height,\;cm\right)\;\; & +\;\left(0.245\;\times \;weight,\;kg\right) \end{array}$$

$$\begin{array}{lll} & TBW\;\left(males\right)=\;2.447\;\;\left(0.09516\times \;age,\;y\right)\;\; & +\;\left(0.1074\;\times \;height,\;cm\right)÷\;\left(0.3352\;\times \;weight,\;kg\right) \end{array}$$

The individual Vd is determined as


$$\begin{array}{l} Vd\;\left(male\right)\;=\;TBW\;÷\;\left(weight,\;kg\;\times \;0.825\right)\;\pm \;9.86\;\%\; \end{array}$$

$$\begin{array}{l} Vd\;\left(female\right)\;=\;\;\;TBW\;÷\;\left(weight,\;kg\;\times \;0.838\right)\;\pm \;15.00\;\%\; \end{array}$$

For the retrograde calculations (back-calculating the BAC from a measured value), the ASB recommends calculating BAC using the range of β values 0.10 to 0.25 g/L/h.

## Case report

A 22-year-old woman (weight, 60 kg; height, 163 cm) worked in a factory. She testified that she had not consumed any food before, during, or after the day of her employment. At 8:30 am, 1 hour before arriving at work, she drank a 12-oz (355-mL) can of 4.65% beer (12.6 g ethanol) and a shot of 49.5% vodka (18 g ethanol). She arrived at work at 9:30 am. No more ethanol was consumed until the noon break, when she consumed 12 oz (22.7 g) of 8.1% beer. At 1:00 pm, she consumed 36 oz (1065 mL) of 8.1% beer and 2 shots of vodka (68.1 g ethanol total). At this point, her coworkers noted signs of alcohol impairment, including slurred speech, unsteady gait, bloodshot eyes, and poor penmanship on reports. At 4:00 pm, the woman drank another 12-oz (355-mL) can of 8.1% beer (22.7 g ethanol). At 4:45 pm, she was evaluated by her supervisor, who instructed her to depart of her own accord. The woman got into her car and drove away. At 6:30 pm, she ran past a stop sign at high speed and collided with another vehicle, killing that vehicle’s driver. Police records show that the woman exhibited signs of alcohol impairment, including an unsteady gait, bloodshot eyes, alcohol on her breath, and slurred speech, and that she failed field sobriety tests. In custody, a breath alcohol taken at 8:30 pm was 2.20 g/L. The woman was charged and convicted of vehicular manslaughter. In court, she provided an account of precisely what brand of alcohol she consumed and when she drank it. She testified that she did not drink after being discharged from work, and no alcohol was found on her person or in her vehicle after the accident. The woman was convicted and sentenced to 10 years in prison.

After the criminal trial, the decedent’s family sued the woman’s employer. The woman’s testimony was used as evidence against her former employer. Because she was incarcerated and therefore there was no issue of self-incrimination, her alcohol consumption history was considered accurate. The plaintiff’s counsel hired an expert witness to provide an estimate of the woman’s BAC at the time of her discharge to assess impairment and concluded that her supervisor was negligent in releasing her. Assuming an accurate drinking history, Table 1 shows what the woman’s theoretical BAC calculations would have been at 4:45 pm, the relevant time in question. Results are calculated using the range of Vd values recommended by the ASB for female individuals, both with and without knowledge of the patient’s TBW. After each alcohol dose, the maximum BAC is computed using the Widmark formula and added to the existing BAC. Following absorption, the BAC is metabolized over time, and calculations are made using the lowest and highest β values. For the theoretical calculation, the extreme range of BACs was between 1.40 and 4.60 g/L (mean, 2.90 g/L). Because the TBW was known, the BAC range was reduced to 1.60 to 3.80 g/L (mean, 2.60 g/L). For the retrospective calculation to the projected BAC at 4:45 pm, the range of BAC results was 2.60 to 3.10 g/L for the 2 β rates that were computed, starting with the measured value of 2.20 g/L at 8:30 pm that day (Table 1). Because the theoretical and retrograde calculations largely matched each other, the toxicology expert opined that the woman was intoxicated at the time of her discharge and that the intoxication was not the result of additional drinking after leaving work. With this information, the defendants in the civil case accepted the settlement with the plaintiff.

**Table 1.** Blood Alcohol Calculations in This Case Report.

	Vd, L/kg^a^	β, g/L/h	BAC, g/L
**Forward calculation: consumption at 8:30** **am** **to work discharge at 4:45** **pm**			
Without TBW	25.8	0.10	4.6^b^
	43.8	0.10	2.4
	25.8	0.25	3.3
	43.8	0.25	1.4^c^
Mean			2.9
With TBW	30.6	0.10	3.8^b^
	41.4	0.10	2.6
	30.6	0.25	2.5
	41.4	0.25	1.6^c^
Mean			2.6
**Back calculation: metabolism from 8:30** **pm** **with a BAC of 2.20 g/L to 4:45** **pm** **(time of employee discharge)**			
		0.10	2.6
		0.25	3.1
Mean			2.8

Abbreviations: BAC, blood alcohol concentration; TBW, total body water; Vd, volume of distribution.

^a^Liters per kilogram of body weight.

^b^Highest calculated value.

^c^Lowest calculated value.

## Discussion

All laboratory measurements, including BAC, have uncertainty. Nevertheless, results of clinical laboratory testing are reported in exact values, excluding the test’s analytical and biological variation. When health care professionals interpret test results, it is assumed that they understand these limitations. In a courtroom, jurists assume that selected jury members do not have the same understanding of test result uncertainty. For this and other reasons, the ASB has recommend against experts reporting calculated BAC results as a single value in favor of reporting a range of values^3^ to account for the variations in human metabolism and tolerance,^4^ and the uncertainty of the facts surrounding the case, especially the alcohol consumption history, which are often misreported.

This case showed that if the alcohol consumption time is prolonged (ie, >8 hours in this case), use of the ASB-recommended Vd and β ranges will produce a wide range of BAC values (especially if the TBW is not known). Although not relevant in this case, a wide range may invite differing legal interpretations, particularly for criminal cases, where the level of evidence must reach a higher standard than for civil cases. Alcohol-tolerant individuals may not be physiologically impaired at a BAC of 1.40 g/L, but a value of 4.60 g/L in an alcohol-naive individual is likely fatal.^5^ Fortunately, in most criminal and civil cases, other evidence is available to enable an appropriate interpretation of BACs.

This case shows that when there is a prolonged period between drinking and some sentinel event, a large range of computed BAC values could insert ambiguity among jurists as they interpret results.
